# Understanding the Factors That Influence the Adoption and Meaningful Use of Social Media by Physicians to Share Medical Information

**DOI:** 10.2196/jmir.2138

**Published:** 2012-09-24

**Authors:** Brian S McGowan, Molly Wasko, Bryan Steven Vartabedian, Robert S Miller, Desirae D Freiherr, Maziar Abdolrasulnia

**Affiliations:** ^1^Education Technology ConsultantBlue Bell, PAUnited States; ^2^Department of Management, Information Systems and Quantitative MethodsUniversity of Alabama at BirminghamBirmingham, ALUnited States; ^3^Baylor College of MedicineBaylor CollegeHouston, TXUnited States; ^4^Breast Cancer ProgramThe Sidney Kimmel Comprehensive Cancer CenterJohns Hopkins Green Spring StationLutherville, MDUnited States; ^5^CE Outcomes, LLCBirmingham, ALUnited States

**Keywords:** Social media, continuing medical education, physicians and social media, physician-physician relationship, oncologists, primary care physicians, education technology, physicians' practice patterns

## Abstract

**Background:**

Within the medical community there is persistent debate as to whether the information available through social media is trustworthy and valid, and whether physicians are ready to adopt these technologies and ultimately embrace them as a format for professional development and lifelong learning.

**Objective:**

To identify how physicians are using social media to share and exchange medical information with other physicians, and to identify the factors that influence physicians’ use of social media as a component of their lifelong learning and continuing professional development.

**Methods:**

We developed a survey instrument based on the Technology Acceptance Model, hypothesizing that technology usage is best predicted by a physician’s attitudes toward the technology, perceptions about the technology’s usefulness and ease of use, and individual factors such as personal innovativeness. The survey was distributed via email to a random sample of 1695 practicing oncologists and primary care physicians in the United States in March 2011. Responses from 485 physicians were analyzed (response rate 28.61%).

**Results:**

Overall, 117 of 485 (24.1%) of respondents used social media daily or many times daily to scan or explore medical information, whereas 69 of 485 (14.2%) contributed new information via social media on a daily basis. On a weekly basis or more, 296 of 485 (61.0%) scanned and 223 of 485 (46.0%) contributed. In terms of attitudes toward the use of social media, 279 of 485 respondents (57.5%) perceived social media to be beneficial, engaging, and a good way to get current, high-quality information. In terms of usefulness, 281 of 485 (57.9%) of respondents stated that social media enabled them to care for patients more effectively, and 291 of 485 (60.0%) stated it improved the quality of patient care they delivered. The main factors influencing a physician’s usage of social media to share medical knowledge with other physicians were perceived ease of use and usefulness. Respondents who had positive attitudes toward the use of social media were more likely to use social media and to share medical information with other physicians through social media. Neither age nor gender had a significant impact on adoption or usage of social media.

**Conclusions:**

Based on the results of this study, the use of social media applications may be seen as an efficient and effective method for physicians to keep up-to-date and to share newly acquired medical knowledge with other physicians within the medical community and to improve the quality of patient care. Future studies are needed to examine the impact of the meaningful use of social media on physicians’ knowledge, attitudes, skills, and behaviors in practice.

## Introduction

The amount of information required for medical practice is growing at an exponential rate, and the ability for one physician to stay completely abreast of the entirety of this knowledge base has long since been surpassed [1]. Physicians in primary care fields and data-intensive specialties such as oncology bear a particularly heavy burden in consuming and managing the amount of information available to them [1-3]. Over the next decade, the cognitive limitation of the traditional model, wherein physicians are expected to learn, retain, and call upon an ever-expanding body of medical knowledge, will become more challenging to navigate. New models for learning and sharing will be needed.

Social learning theory has long been applied to medical education [2,4,5]. In the past, these explorations focused on simple connections derived from training pedigree, geography, and shared memberships in medical societies or associations, and connectedness was largely episodic (eg, annual meetings, committee work, and listservs). However, with the emergence of social media, the concept of social learning can encompass a myriad of nontraditional connections and uses.

Social media websites and applications are online environments where users contribute, retrieve, and explore content primarily generated by fellow users. As opposed to more traditional forms of information and communication technologies used in health care organizations, the content generated through social media is typically created by users for users, thus allowing knowledge and support to flow more effectively through a professional social network, and allowing answers and support to be more effectively leveraged across a professional social network [6-9].

Despite a growing body of literature highlighting both the promises and controversies associated with social media usage in health care, there are surprisingly few empirical studies on those most affected by it—physicians themselves [10-12]. There is also variation in how social media were selected, perceptions were collected, and usage was examined, thus limiting the value of conclusions [13,14]. For instance, one recent survey of the literature identified 46 unique definitions of social media across 44 articles of Health 2.0 and Medicine 2.0 publications [15]. Additionally, while patients are embracing social media technologies to share information with other patients and health care experts, practicing physicians seem to be more reluctant to move into a new age of collaborative health care [16].

We used a theoretical framework for assessing and predicting the adoption of social media by physicians for the specific use of sharing medical knowledge and lifelong learning, and explored whether adoption differs between two specialties—primary care and oncology—in rapidly changing medical knowledge environments. We developed the research model based on the Technology Acceptance Model (TAM) (Multimedia Appendix 1), hypothesizing that technology usage is best predicted by a physician’s attitudes toward the technology, perceptions about the technology’s usefulness and ease of use, and individual factors such as personal innovativeness and beliefs.

## Methods

### Study Design

We conducted a cross-sectional study of physicians in the fields of primary care and oncology who practice in the United States to test the following primary hypothesis: physicians who perceive social media as easy to use and useful and who have positive attitudes toward its use are more likely to share medical knowledge with other physicians through social media. This protocol was approved by the Western Institutional Review Board (Olympia, WA, USA).

### Model, Measures, and Data Collection

**Figure 1:**
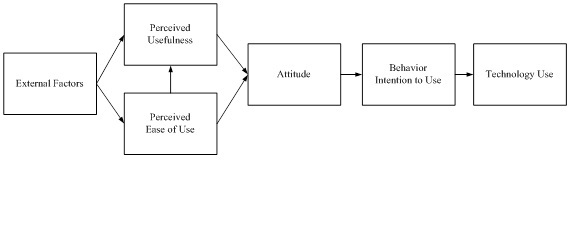
The Technology Acceptance Model predicts that ease of use and usefulness will influence an individual’s attitudes towards, intention to use, and acceptance of the technology.

We designed the survey to test the theoretical framework posited in TAM [17]. TAM proposes that an individual’s acceptance of a technology is determined by its perceived usefulness and perceived ease of use. The model predicts that ease of use and usefulness will influence an individual’s attitudes toward, intention to use, and acceptance of the technology (Figure 1) [17].

 Survey questions were adapted from previously published scales. We field tested the survey instrument for clarity and comprehensiveness with 2 physicians in the intended target audience prior to implementation. Using multi-item scales for each construct, the survey assessed (1) the perceived barriers to social media adoption, (2) motivations to adopt social media, including desire to advance the profession, personal innovativeness, and access to peers, (3) attitudes toward social media, (4) perceived ease of use of social media, (5) perceived usefulness of social media, and (6) usage of social media to share medical knowledge with other physicians. The final instrument included 27 items assessing the constructs of interest. Response categories for barriers, motivations, perceived ease of use, and perceived usefulness (independent variables) consisted of a 7-point scale ranging from strongly disagree to strongly agree. Attitudes toward social media usage were assessed using 10-point semantic differential scales. The outcome was the frequency of use of social media to share medical knowledge with other physicians (dependent variable). Response categories for current frequency of use were never, rarely, monthly, once a week, 3 times a week, daily, and many times a day (Multimedia Appendix 1).

For this study, we defined social media as Internet-based applications that allow for the creation and exchange of user-generated content, including services such as social networking, professional online communities, wikis, blogs, and microblogging. We defined use as the exchange of information, advice, ideas, reports, and scientific discoveries with other physicians in the medical community. Additional questions were used to understand adoption on a social media application-specific basis. We did not use these data in the TAM analyses, but to provide a more granular perspective on current levels of use and future intention to use each application.

A national sample of 1695 physicians was randomly selected from the American Medical Association’s Physician Masterfile: 699 were practicing in oncology and 996 were practicing in primary care. We sent an email invitation to all physicians in the sample in March 2011 to participate in the survey. An honorarium of US $50 was offered for completing the survey.

### Analysis

Prior to performing hypothesis tests, we assessed the reliability and validity of the survey items. To assess reliability, which indicates the degree of agreement between the multiple items making up a construct, we determined the Cronbach alphas for our variables. The average Cronbach alpha was .92, and all constructs were higher than the recommended threshold of .70. Discriminant validity is useful to demonstrate the extent to which a construct of interest differs from others. To assess discriminant validity, we used a principal component factor analysis to test that the various items loaded highest on their theoretical constructs. We ran a 1-factor analysis containing all multi-item constructs using varimax rotation and extracting 8 factors; all items loaded on their expected factors at a level of .69 or higher, indicating adequate discriminant validity.

We used hierarchical regression analysis to test the theoretical model. Hierarchical regression analysis makes it possible to test whether a set of variables, entered as a block and in a theoretically justified order, adds significantly to variance already explained by a prior set of variables. In the first step, we entered demographic variables. In the second step, we entered the individual factors (barriers, motivations to advance the professional community, personal innovativeness, and peer access). In the third step, we entered attitudes toward social media, perceived ease of use, and perceived usefulness. The specified order of entry allows the rigorous testing of the effects of individual factors over and above the demographic variables, as well as the impact of attitudes and perceptions above all the previously entered factors (Multimedia Appendix 2).

To assess the severity of multicolinearity, which occurs when 2 or more predictor variables in a multiple regression are highly correlated, we calculated the variance inflation factor for each variable. All variance inflation factors were below the generally accepted 5.0 cut-off. We also performed the analysis dropping the usefulness variable from the regressions. The amount of variance explained in the final model dropped from .57 to .52, and the pattern of results was the same. We therefore included the usefulness variable in our final model. In reporting the data, we used *P *= .05, 2-sided, as the criterion for statistical significance of the estimated effects of the independent variables on the frequency of social media usage by physicians. All analyses were performed with PASW statistics software, version 18 (IBM Corporation, Somers, NY, USA).

## Results

We received responses from 491 of the 1695 physicians contacted, resulting in a response rate of 28.97%. However, 6 respondents self-classified as nonpracticing physicians were removed and a sample of 485 was analyzed (186 oncologists and 299 primary care physicians; Table 1). To assess response bias, we compared the results on key attitudinal questions and demographic variables between early and late responders. There were no significant differences between early and late responders, minimizing the concern of response bias in our sampling frame. To assess nonresponse bias, we compared the demographics of our sampling frame with the overall demographics of primary care physicians and oncologists in the United States and found no discernible differences, minimizing the threat of nonresponse bias.

**Table 1:** Sample characteristics.

		Oncology (n = 186)	Primary care (n = 299)
Degree (MD/DO), n (%)		186 (100.0%)	299 (100.0%)
Male gender, n (%)		140 (75.3%)	216 (72.2%)
Years since medical school graduation, mean (SD)		24 (10)	24 (9)
**Practice location, n (%)**			
	Urban	88 (47.3%)	70 (23.4%)
	Suburban	82 (44.1%)	179 (59.9%)
	Rural	17 (9.1%)	50 (16.7%)
**Practice setting, n (%)**			
	Solo	23 (12.4%)	102 (34.1%)
	Group	128 (68.8%)	178 (59.5%)
	Medical school	17 (9.1%)	3 (1.0%)
	Nongovernment hospital	9 (4.8%)	8 (2,7%)
**Major professional activity, n (%)**			
	Direct patient care	181 (97.3%)	292 (97.7%)
	Other	5 (2.7%)	7 (2.3%)

**Figure 2:**
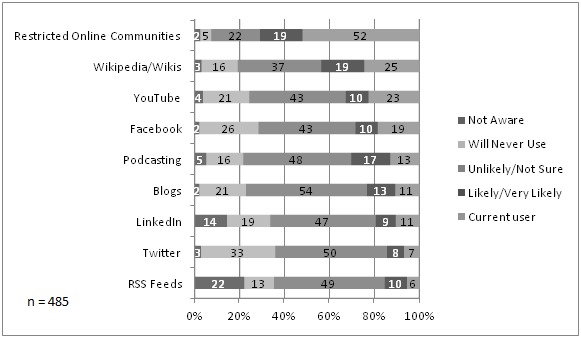
Respondents' current use and intention to use social media.

The study was designed to understand at a granular level the current adoption and the intent to adopt social media for the exchange of information, advice, ideas, reports, and scientific discoveries with other physicians in the medical community. Figure 2 shows what applications and platforms were being used by respondents. By providing a list of specific applications within the survey, our intent was to underscore the broader definition of social media used within the TAM analyses. Across all applications, awareness was high with 78%–98% of respondents claiming to be aware of the application. Current use varied on an application-specific basis from 33 of 485 (6.8%) for Twitter to 252 of 485 (52.0%) for online physician-only communities (such as Sermo, Ozmosis, or medical society membership sites). For each application there was a subset of respondents (between 5% and 33%) who claimed that they “will never use” the application for the exchange of information, advice, ideas, reports, and scientific discoveries with other physicians in the medical community. But for most applications (except restricted online communities), the largest portion of the respondents identified themselves as currently unlikely or unsure about their intent to use.

**Figure 3:**
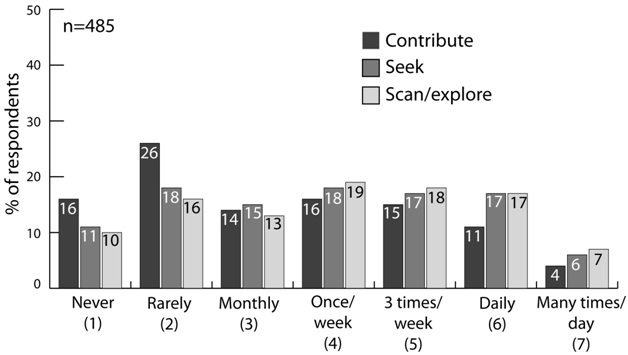
Physicians' frequency of using social media to contribute medical knowledge to other physicians, to seek specific information about a medical problem or situation, and to scan or explore medical knowledge for new insights.

Figure 3 shows the frequency distribution of social media usage. Respondents indicated how frequently they (1) were using social media to *contribute *medical knowledge to other physicians, (2) were using social media to *seek *specific information about a medical problem or situation, and (3) were using social media to *scan or explore *medical knowledge for new insights. Overall, 117 of 485 (24.1%) of respondents used social media daily to scan or explore medical information, whereas 69 of 485 (14.2%) contributed new information via social media on a daily basis. These numbers rose to 296 of 485 (61.0%) scanning and 223 of 485 (46%) contributing once a week or more.

**Figure 4:**
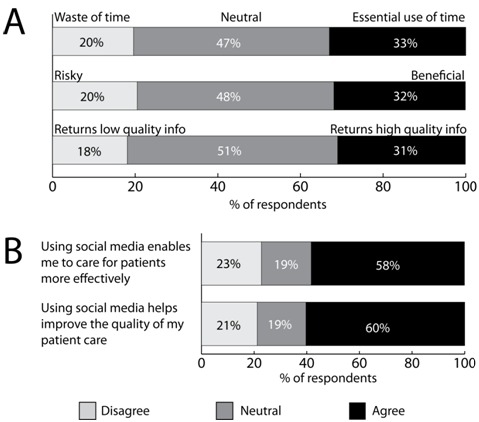
Respondents expressed how they felt about the use of social media along 3 dimensions: perceived risk, perceived usefulness, and perceived quality of information (part A). Part B shows how respondents perceived their engagement and use of social media to affect their competency and clinical performance. n = 485.

Among the variables that TAM explores is the general attitudes that respondents have toward the usefulness of social media for the exchange of information, advice, ideas, reports, and scientific discoveries with other physicians in the medical community. Figure 4 (part A) shows how respondents felt about the use of social media along 3 dimensions: perceived risk, perceived usefulness, and perceived quality of information. Approximately one-third of respondents found social media to be an essential use of time, to be beneficial, and to return high-quality information. Figure 4 (part B) shows how respondents perceived their engagement and use of social media to affect their competency and clinical performance. Approximately 60% of respondents (281 of 485) stated that social media enabled them to care for patients more effectively and improved the quality of patient care they delivered (291 of 485).

Table 2 shows the correlations between constructs and the variance inflation factors. Although the majority of correlations were modest, 2 variables were very strongly correlated with the perceptions of usefulness of the technology: attitudes toward usage (.80) and frequency of usage (.72). These variables indicate that respondents who had strong positive attitudes about using social media and had found using social media to be useful in enhancing their performance and patient care were significantly more likely to be frequent users of social media.

**Table 2:** Correlation between variables and variance inflation factors (VIFs).

Variable		Specialty	Year	Gender	Patients/ week	Barriers	Advance community	Innovativeness	Peer access	Attitudes	Ease of use	Usefulness	VIF
**Specialty**												1.064	
	*P *value												
**Graduation year**		.017											1.11
	*P *value	.71											
**Gender**		.027	–.16										1.093
	*P *value	.55	.001										
**Patients per week**		.177	.021	–.134									1.066
	*P *value	.000	.65	.003									
**Barriers**		–.047	.114	.014	.047								1.417
	*P *value	.30	.01	.75	.42								
**Advance the professional community**		.010	–.056	.018	.004	–.210							1.223
	*P *value	.83	.22	.69	.92	.000							
**Personal innovativeness**		–.090	–.112	–.124	.056	–.201	.305						1.524
	*P *value	.047	.01	.006	.22	.000	.000						
**Peer access**		.035	–.141	.052	.023	–.408	.316	.484					2.509
	*P *value	.44	.002	.26	.61	.000	.000	.000					
**Attitudes**		.078	–.165	.084	.048	–.344	.350	.426	.689				3.109
	*P *value	.09	.000	.07	.29	.000	.000	.000	.000				
**Ease of use**		–.009	–.274	.038	.048	–.491	.342	.418	.502	.474			1.811
	*P *value	.85	.000	.41	.29	.000	.000	.000	.000	.000			
**Usefulness**		.043	–.160	.047	.030	–.384	.343	.463	.726	.802	.493		3.426
	*P *value	.35	.000	.30	.51	.000	.000	.000	.000	.000	.000		
**Frequency of use**		.026	–.121	.075	.064	–.274	.280	.439	.638	.661	.478	.718	
	*P *value	.57	.007	.10	.16	.000	.000	.000	.000	.000	.000	.000	

Table 3 shows results from the hierarchical regression analysis. In the first step that included the demographic variables, the only significant predictor of usage was years since medical school, indicating the physicians who were younger were likely to use social media more frequently; however, the amount of variance explained was less than 2%. The control variable for specialty was not significant, indicating that there were no significant differences in the frequency of usage patterns between oncologists and primary care physicians. In step 2, we added the barriers and individual factors to the model. The amount of variance explained in the frequency of usage increased to 43%, with the variables personal innovativeness and gaining access to influential peers being the key predictors. Age was no longer significant, however, but gender became significant in step 2.

In step 3, the final model, we explained 57% of the variance in frequency of usage. The demographic variables were no longer significant. Barriers, which was not significant in the first 2 steps, became significant but in a positive direction. This is surprising given that barriers to use has a significant negative bivariate correlation with usage frequency, and intuitively we expected that the higher the barriers to usage, the less frequently physicians would use social media. This indicates that once attitudes toward social media and perceptions of its usefulness and ease of use are taken into account, respondents frequently use social media even though the perceived barriers are high. The ability to gain access to influential peers remained significant, indicating that respondents would use social media more frequently because they are motivated by accessing learning and decision-making resources based on the collective knowledge of their peers. Positive attitudes toward social media usage and perceptions about its ease of use and usefulness were also significant predictors of usage frequency.

Although specialty was not significant in the hierarchical regression model, one of the goals of this research was to determine whether there were differences not only in the frequency of social media usage, but also in the predictors explaining usage frequency. These data suggest that oncologists are more likely to be influenced by motivations of personal innovativeness, while primary care physicians are more likely to be influenced by having access to peers (Table 3). Both groups were influenced by positive attitudes toward social media, ease of use, and usefulness.

**Table 3:** Hierarchical regression results (standardized beta).

Variable	Step 1: Demographics		Step 2: Barriers and motivations		Step 3: Full model		Oncologists		Primary care	
	Beta	*P *value	Beta	*P *value	Beta	*P *value	Beta	*P *value	Beta	*P *value
Specialty	.019	.69	.014	.70	–.003	.92	NA^a^	NA	NA	NA
Graduation year	–.108	.02	–.012	.74	.039	.21	0.076	.16	0.008	.83
Gender	.067	.15	.074	.04	.046	.15	0.058	.28	0.026	.50
Patients per week	.073	.12	.050	.16	.033	.29	0.013	.80	0.040	.29
Barriers			–.013	.72	.083	.02	0.110	.08	0.060	.16
Advance the professional community			.058	.12	–.022	.50	–0.103	.06	0.027	.53
Personal innovativeness			.171	.000	.070	.06	.133	.02	0.006	.90
Peer access			.523	.000	.169	.000	0.061	.43	.254	.000
Attitudes					.153	.004	.178	.04	.143	.04
Ease of use					.154	.000	.247	.000	.105	.04
Usefulness					.407	.000	.412	.000	.384	.000
*R* *2 *adjusted	.015	.03	.428	.000	.567	.000	.529	.000	.594	.000
Change in *R* *2*			0.415	.000	.139	.000				

## Discussion

As the amount of medical knowledge required for patient care continues to expand, social media technologies may provide an efficient and effective tool for educating and informing practicing physicians. Our findings suggest that although a small percentage of respondents were using social media on a daily basis to seek, scan, or contribute medical knowledge with other physicians, up to half of the respondents were taking these actions on a weekly basis. The portion rose to more than 70% of respondents using social media on at least a monthly basis.

From this study sample, it appears that the frequency of social media usage is influenced primarily by positive attitudes toward the technology, perceiving that the technology is easy to use, and perceiving the technology to be useful to achieve better performance outcomes. Conversely, factors found to be nonsignificant included demographic variables typically perceived as important, such as years since graduation (a proxy for age), gender, patients seen per week (a proxy for how busy a physician is), and type of specialty. This finding is consistent with other studies, which have shown practice-related characteristics to be unassociated with use of Internet-based communication technologies [14].

Social media usage was clearly application specific and, once we moved beyond the general definition of social media that was used in the TAM analyses, we found a 6- to 7-fold variation in the extent of adoption across the list of applications we explored. Interestingly, more than half of respondents had adopted online physician-only communities for the purpose of exchanging medical information with other physicians. While future studies should explore the predictors and barriers to adoption at an application-specific level, it stands to reason from these data that the elements of TAM themselves are application specific. For example, respondents might see online communities as a less-risky and higher-quality source of medical knowledge than more broadly open social media applications such as Twitter, LinkedIn, or Facebook.

In this study sample, one key difference between oncologists and primary care physicians was the underlying factors influencing usage. Oncologists were more likely to be motivated to use social media out of a sense of personal innovativeness. This could, in part, be due to a characteristic of the professional culture of oncologists regarding a perceived need to be on the cutting edge of science and clinical practice [18,19]. In contrast, primary physicians were more likely to be motivated to use social media out of a need to have access to and be influenced by peer physicians [20]. In addition to individual factors and attitudes, respondents were far more likely to use social media to share medical knowledge with other physicians when they perceived that learning the technology was easy for them and when it resulted in useful performance outcomes, such as increased practice efficiency and enhanced patient outcomes. Furthermore, though perceived barriers were high, respondents were still willing to use social media more frequently if they have found it to be useful. These findings indicate that, for now, the key factors influencing frequent usage are experiential factors, and they are achieved only after initial adoption of the technology and a period of usage. But it is important to note that there are no definitive studies demonstrating that the use of social media for the exchange of medical information with other physicians as a component of their lifelong learning and continuing professional development leads to more learned physicians or better patient outcomes. For now, personal experience and anecdotes are likely to be the primary drivers of these positive attitudes.

Perhaps, as physicians increasingly experiment with social media technologies, these tools may provide an efficient and effective means for staying abreast of the vast amount of medical knowledge required to deliver patient care. This might be transformative in medicine, as traditional lecture-based continuing medical education has been shown to be largely ineffective in changing physician behavior at the same time that medical knowledge is changing at the fastest pace in history [21-23]. Social media technologies could complement (or even replace) continuing medical education for physicians as either an informal or formal learning channel [24-26]. But for now, how social media channels are the vehicles through which physicians are exposed to emerging information that has the potential to inform or change practice remains an open question [27,28].

As one of the first studies to examine the factors that influence the frequency of social media usage by physicians to share medical knowledge with other physicians, this study has several strengths and limitations. The first strength of this research was establishing clear definitions for our key constructs of interest, specifically definitions for what constitutes social media and clarifying the type of usage of interest (to share medical knowledge with other physicians). The second important strength was the grounding of this research in theory based on TAM, and the use of previously validated, multi-item survey scales to ensure the reliability and validity of the findings. Third, this survey studied two medical specialties characterized by a rapidly changing and dynamic medical knowledge base.

However, limitations of the study include a narrow focus on two medical specialties and one specific definition of usage; a need for a better understanding of the barriers to using social media for lifelong learning; and a need for a better understanding of why respondents indicated they would never use certain channels to exchange medical advice with other physicians. It should be noted that the first limitation is closely related to one of the strengths of this study: the definition of usage was narrowly focused on sharing medical knowledge with other physicians. Therefore, our findings are not generalizable to other types of usage: the use of social media by physicians to treat or to educate patients; nor are they generalizable to physicians’ personal use. In addition, there is little prior research directing our exploration of the barriers to use as they uniquely relate to the exchange of medical information with other physicians as a component of their lifelong learning and continuing professional development. Therefore, this study relied on more general barriers, including risks related to privacy, access to social media applications in practice, and time available to use and explore these technologies. Our predictive analyses probably would have yielded even stronger results had we had a more robust understanding of use-specific barriers.

Future studies should examine potential differences between other populations of physicians and other types of health care professionals (specifically, rural and urban; and emergency professions and public health professionals) in terms of their use of social media to share and exchange medical knowledge. Studies should also examine different types of social media usage beyond the sharing of knowledge with other physicians.

### Conclusions

The amount of information that a practicing clinician must learn, understand, and apply in practice is growing at unprecedented levels and has long surpassed our cognitive capacities. Social media and social learning models in general provide an important opportunity to manage this information overload, but only if the media are being used effectively. This study demonstrates that the adoption of social media to exchange information and medical knowledge with other physicians is strongly dependent on the perceived usefulness of the technology and the general attitudes physicians have toward the value these technologies offer. Efforts should be made to further explore these predictors of use. These follow-up studies must be conducted with rigor and must move the science of professional learning and development forward in discernible steps to allow physicians to fully embrace a collaborative approach to care.

## Multimedia Appendix 1

Construct definitions.

## Multimedia Appendix 2

Survey Instrument.

## Abbreviations

TAM: Technology Acceptance Model
