# Supplementary material for: Understanding the Factors That Influence the Adoption and Meaningful Use of Social Media by Physicians to Share Medical Information
Source: J Med Internet Res. 2012 Sep 24;14(5):e117. doi: 10.2196/jmir.2138 (PMC3510763; doi:10.2196/jmir.2138)
Supplement: Supplementary file 1 [file jmir_v14i5e117_app1.pdf]

Appendix 1: Construct Definitions

Construct	Definition	Sample Survey Item	Scale
Frequency of Social Media Usage	The overall frequency of physician usage of social media applications in general to contribute, retrieve and explore medical knowledge with other physicians	What is your overall frequency of using social media to **contribute medical knowledge** to other physicians?	1-7; Never to Many times a day
Attitudes toward Social Media Usage	A person's perspective towards the usage of social media applications to share medical knowledge with other physicians	A bad idea… A good idea	1-10; Semantic Differential
Usefulness	A person's perspective towards the utility of social media applications to share medical knowledge with other physicians	Using social media enables me to care for patients more effectively	1-7; Strongly disagree to Strongly agree
Ease of Use	A person's perspective towards the ease of use of social media applications to share medical knowledge with other physicians	Learning to use social media was easy for me	1-7; Strongly disagree to Strongly agree
Personal Innovativeness	A person's attitudes towards innovating with social media	I actively seek new ways to use social media in my practice	1-7; Strongly disagree to Strongly agree
Advance the Profession	A person's desire to advance the profession as a whole	I feel that it is important to help others to advance the professional community	1-7; Strongly disagree to Strongly agree
Access to Peers	People who are important to responder encourage the use of social media	There is a strong physician community that I can access using social media	1-7; Strongly disagree to Strongly agree
Barriers	Impediments detracting from the use of social media	I don't have time to learn how to use social media for professional purposes	1-7; Strongly disagree to Strongly agree
