# Supplementary material for: Understanding the Factors That Influence the Adoption and Meaningful Use of Social Media by Physicians to Share Medical Information
Source: J Med Internet Res. 2012 Sep 24;14(5):e117. doi: 10.2196/jmir.2138 (PMC3510763; doi:10.2196/jmir.2138)
Supplement: Supplementary file 2 [file jmir_v14i5e117_app2.pdf]

Appendix 2: Survey Instrument

For the purpose of this study, we are interested in how physicians are using social media to share medical knowledge with other physicians.

Social media is defined as internet-based applications which allow for the creation and exchange of user-generated content and includes services such as social networking, professional online communities, wikis, blogs, and microblogging.

Sharing medical knowledge refers to the exchange of information, advice, ideas, reports and scientific discoveries with other physicians in the medical community.

Current Use and Intent to Use Social Media

	Not Aware (1)	Will Never Use (2)	Unlikely /Not Sure (3)	Likely / Very Likely (4)	Current User (5)
Restricted Online Communities	☐	☐	☐	☐	☐
Wikipedia/Wikis	☐	☐	☐	☐	☐
YouTube	☐	☐	☐	☐	☐
Facebook	☐	☐	☐	☐	☐
Podcasting	☐	☐	☐	☐	☐
Blogs	☐	☐	☐	☐	☐
LinkedIn	☐	☐	☐	☐	☐
Twitter	☐	☐	☐	☐	☐
RSS Feeds	☐	☐	☐	☐	☐

Frequency of Social Media Usage

	Never (1)	Rarely (2)	Monthly (3)	Once a week (4)	3 times a week (5)	Daily (6)	Many times a day (7)
What is your overall frequency of using social media to **contribute** medical knowledge to other physicians?	□	□	□	□	□	□	□
What is your overall frequency of using social media to **seek specific information** about a medical problem/situation?	□	□	□	□	□	□	□
What is your overall frequency of using social media to **scan/explore** medical knowledge for new insights?	□	□	□	□	□	□	□

Attitudes toward Social Media Usage

When sharing medical advances and knowledge with other physicians, using social media is _____________.

Please indicate where you fall along the following dimensions in your attitude towards the use of social media.

1 2 3 4 5 6 7 8 9 10

A waste of time An essential use of time

1 2 3 4 5 6 7 8 9 10

Very risky Very beneficial

1 2 3 4 5 6 7 8 9 10

Boring Very Engaging

1 2 3 4 5 6 7 8 9 10

A bad way to get current info A great way to get current info

1 2 3 4 5 6 7 8 9 10

Returns low quality information Returns high quality information

Usefulness

**Using Social Media…**	Strongly Disagree	Disagree	Somewhat Disagree	Neither Agree or Disagree	Somewhat Agree	Agree	Strongly Agree
… enables me to accomplish job tasks more effectively.	☐	☐	☐	☐	☐	☐	☐
… improves my job performance.	☐	☐	☐	☐	☐	☐	☐
… increases my job productivity.	☐	☐	☐	☐	☐	☐	☐
…enables me to care for patients more effectively.	☐	☐	☐	☐	☐	☐	☐
… helps improve the quality of my patient care.	☐	☐	☐	☐	☐	☐	☐

Ease of Use

	Strongly Disagree	Disagree	Somewhat Disagree	Neither Agree or Disagree	Somewhat Agree	Agree	Strongly Agree
Learning to use social media was easy for me.	☐	☐	☐	☐	☐	☐	☐
It was easy for me to become skillful at using social media.	☐	☐	☐	☐	☐	☐	☐
I find social media easy to use.	☐	☐	☐	☐	☐	☐	☐

Personal Innovativeness

**Statement**	Strongly Disagree	Disagree	Somewhat Disagree	Neither Agree or Disagree	Somewhat Agree	Agree	Strongly Agree
I actively seek new ways to use social media in my practice	☐	☐	☐	☐	☐	☐	☐
I usually find out about new social media applications	☐	☐	☐	☐	☐	☐	☐

earlier than others							

Advance the Profession

	Strongly Disagree	Disagree	Somewhat Disagree	Neither Agree or Disagree	Somewhat Agree	Agree	Strongly Agree
I feel that it is important to help others to advance the professional community	☐	☐	☐	☐	☐	☐	☐
It’s important to help my professional community succeed	☐	☐	☐	☐	☐	☐	☐
When people share knowledge, the entire professional community benefits	☐	☐	☐	☐	☐	☐	☐

Access to Peers

**Statement**	Strongly Disagree	Disagree	Somewhat Disagree	Neither Agree or Disagree	Somewhat Agree	Agree	Strongly Agree
There is a strong physician community that I can access using social media	☐	☐	☐	☐	☐	☐	☐
There is a critical mass of other physicians that I can reach using social media	☐	☐	☐	☐	☐	☐	☐
People in my profession who are important to me encourage the use of social media	☐	☐	☐	☐	☐	☐	☐
People in my profession who have an influence on me encourage the use of social media	☐	☐	☐	☐	☐	☐	☐

Barriers

	Strongly Disagree	Disagree	Somewhat Disagree	Neither Agree or Disagree	Somewhat Agree	Agree	Strongly Agree
I am too busy to participate in social media	☐	☐	☐	☐	☐	☐	☐
I don't have time to learn how to use social media for professional purposes	☐	☐	☐	☐	☐	☐	☐
I am concerned that using social media will consume too much time once I get started	☐	☐	☐	☐	☐	☐	☐

4. **Approximately how many patients do you see each week? ___________________/ per week**

5. **What year did you graduate from medical school? _____________**

6. **Did you attend medical school in the United States?**	☐	Yes	☐ No
☐ Male ☐ Female			
7. **Are you**			
8. **<u>Degree</u>**	9.	10. **<u>Practice Location</u>**	11. **<u>resentEmployment</u>**
☐ MD/DO	☐ O	☐ Urban	☐ Solo

	ncology		Practice
☐ Other _______	☐ Primary Care	☐ Suburban	☐ Group Practice
	☐ Other __________	☐ Rural	☐ Medical School
			☐ HMO
			☐ Non-Government Hospital
12. **Major Professional Activity**			☐ Government
☐ Direct Patient Care Activities			☐ Other____________
☐ Administrative Activities			
☐ Medical Education			

☐ Medical Research
☐ Other ______________________
